# Untargeted metabolomics identifies a bacterial cyclic dipeptide that induces resistance to a rust fungus of beans

**DOI:** 10.1038/s41598-025-33515-4

**Published:** 2025-12-30

**Authors:** Bret Cooper, Ronghui Yang, Kimberly B. Campbell

**Affiliations:** Soybean Genomics and Improvement Laboratory, USDA-ARS, Beltsville, 20705 MD USA

**Keywords:** Halo blight, Genistein, Daidzein, Phytoalexin, Disease resistance, Immunity, Plant immunity, Secondary metabolism, Metabolomics, Metabolomics

## Abstract

Genistein and daidzein are isoflavonoid phytoalexins that increase rapidly during bean hypersensitive immunity to *Pseudomonas savastanoi* pv. *phaseolicola.* To understand how genistein and daidzein affect *P. savastanoi* pv. *phaseolicola*, non-targeted metabolomic mass spectrometry was performed on the bacterium treated in vitro. The bacterium catabolized genistein and daidzein and responded by producing several different classes of compounds including auxin-like indoles and cyclic dipeptides. Non-targeted metabolomic investigation of bean leaves infiltrated with the cyclic dipeptides revealed no similarities to auxin-induced metabolic changes, but one cyclic dipeptide, cyclo-Trp-Pro (cWP), induced the accumulation of phytoalexins. This implied that cWP application might make beans more resistant to pathogens. Challenge with *Uromyces appendiculatus*, a rust fungal pathogen, revealed that beans pretreated with cWP had 90% reductions in disease. *Arabidopsis thaliana* sprayed with cWP had activated salicylic acid-mediated immune responses. Overall, these results reveal that *P. savastanoi* pv. *phaseolicola* is adapted to tolerate bean genistein and daidzein, likely sensing the compounds as host signals and producing cyclic dipeptides in response. In turn, beans respond to at least one cyclic dipeptide, cWP, by producing phytoalexins to increase resistance. cWP may be useful for protecting beans and other plants from microbial disease.

**Supplementary Information:**

The online version contains supplementary material available at 10.1038/s41598-025-33515-4.

## Introduction

Phytoalexins are antibiotics deployed by plants during immune responses to pathogens^1^. Isoflavonoid phytoalexins, e.g. glyceollin, genistein, daidzein, coumestrol, and related stilbenoids like resveratrol, are derived from phenylalanine and are commonly found in legumes^2,3^. By contrast, the phytoalexin camalexin is an indole derived from tryptophan and is commonly found in crucifers^1^. Pioneering research established that phytoalexins purified from plants inhibited the replication of cellular pathogens in vitro^2^. Subsequent genetics and molecular biology studies established the roles of phytoalexins *in planta.* For example, *Arabidopsis thaliana pad3* mutants deficient in camalexin biosynthesis are more susceptible to *Alternaria brassicicola*^4,5^. Suppression of isoflavone synthase in soybean led to decreased accumulation of glyceollin and decreased resistance to *Phytophthora sojae*^6^. Transgenic tobacco engineered to produce more resveratrol had increased resistance to *Botrytis cinerea*^7^, and transgenic alfalfa expressing isoflavone 7-O-methyltransferase produced more phytoalexins and had increased resistance to *Phoma medicaginis*^8^. These findings helped codify the phytoalexin theory of plant immunity whereby phytoalexins stop disease progression due to their antibiotic activity.

Genistein and daidzein are the two main isoflavonoid phytoalexins produced in bean leaves after infection with *Pseudomonas savastanoi* pv. *phaseolicola*, the bacterium that causes halo blight^3^. Genistein and daidzein are made from 2,4’,5,7-tetrahydroxyisoflavanone and 2,4’,7-trihydroxyisoflavanone, respectively, and diverse phytoalexins and their glycosylated forms are subsequently produced from genistein and daidzein by divergent enzymatic pathways^9^. Daidzin, coumestrol, glyceollin, medicarpin, and pisatin, for example, are derived from daidzein while genistin and prunetin are derived from genistein. Chemically, genistein is hydroxylated daidzein^10^, yet this structural difference could provide unique biochemical activities. In human and mammalian cell systems, genistein acts as a tyrosine kinase inhibitor, but daidzein does not^11–14^.

Genistein slows the growth of pathogenic and enteric bacteria of mammals in vitro, but greater concentrations are needed to slow the bean-adapted *P. savastanoi* pv. *phaseolicola*^3,15–17^. In our ongoing research to understand how phytoalexins biochemically affect bacteria^18–20^, we found that *P. savastanoi* pv. *phaseolicola* can enzymatically degrade genistein^21^. This likely explains why *P. savastanoi* pv. *phaseolicola* suffers fewer detrimental proteomic changes from genistein exposure compared to salicylic acid and resveratrol which are more toxic^19,20^. But not only does *P. savastanoi* pv. *phaseolicola* tolerate genistein by degrading it, the bacterium responds by producing varied indole alkaloids that might exert hormonal functions in beans to favor bacterial pathogenicity^21^. These observations imply that genistein could be a pathogenic trigger for *P. savastanoi* pv. *phaseolicola* infection. Such a possibility would be consistent with the findings that genistein induces *nod* gene expression to enable rhizobium symbiosis with soybean roots and with the observation that genistein induces *P. sojae* (an oomycete) pathogenicity of soybean roots^22,23^. Daidzein also induces *nod* gene expression and *P. sojae* pathogenicity despite the hydroxylation difference^22,23^. Thus, we sought to investigate how *P. savastanoi* pv. *phaseolicola* responded metabolically to daidzein versus genistein. We found that both genistein and daidzein were modified and degraded and that exposure to both phytoalexins induced the bacterial production of indoles and cyclic dipeptides. We discovered that one of the cyclic dipeptides increased the production of daidzein, genistein, and other phytoalexins when applied back to beans. The increase in phytoalexins after cyclic dipeptide application was sufficient to enhance bean resistance to fungal infection.

## Results

### Genistein and daidzein alter small molecule accumulation in a bacterial pathogen

**Fig. 1:**
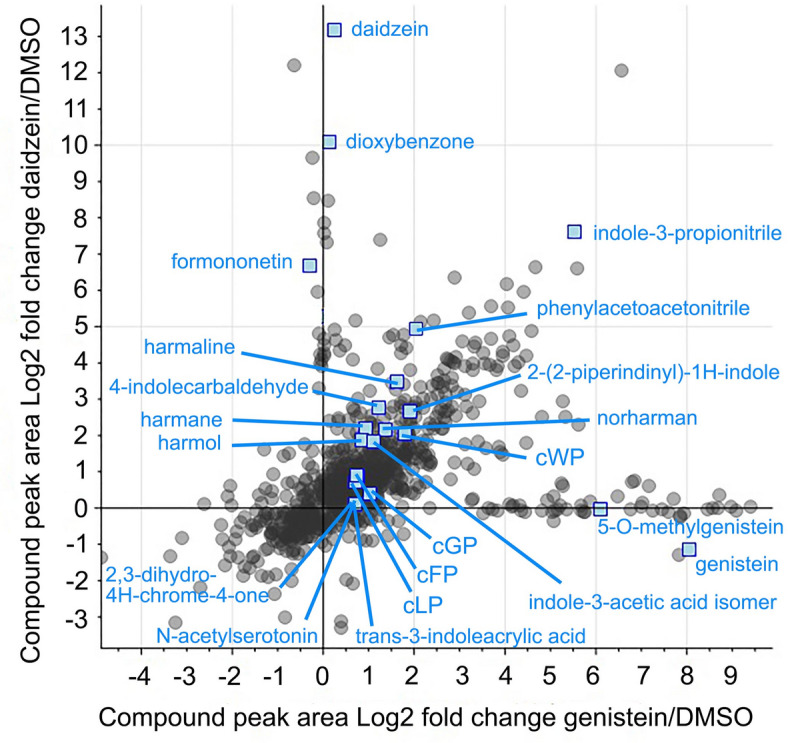
Compounds found in extracts of *P. savastanoi* pv. *phaseolicola* R5 exposed to 400 µM genistein versus 400 µM daidzein (each compared to DMSO controls). The compounds (gray circles) were detected by non-targeted mass spectrometry and their abundances calculated from their chromatographic peak areas. Selected compounds with statistically significant accumulation changes are denoted with blue squares and lettering (FDR < 5%, *n* = 5). cFP, cyclo-Phe-Pro; cGP, cyclo-Gly-Pro; cLP, cyclo-Leu-Pro; and cWP, cyclo-Trp-Pro.

Avirulent *P. savastanoi* pv. *phaseolicola* race 5 (R5) induces hypersensitive immunity on *Phaseolus vulgaris* Black Valentine bean leaves while virulent race 8 (R8) produces water-soaking disease^24^. At the sites of inoculation with either race, the leaf cells accumulate genistein, daidzein, and other phytoalexins, but greater amounts can accumulate during hypersensitive immunity to R5 ^3^. This is because the avirulent bacterium triggers immune system amplification whereas the virulent bacterium suppresses the immune system. Because R5 and R8 are exposed to many different phytoalexins in addition to genistein and daidzein in bean leaves, it is not possible to know how much any single phytoalexin contributes to immunity. Thus, we have used in vitro, liquid growth assays to assess the individual effects of phytoalexins on *P. savastanoi* pv. *phaseolicola* and estimated the minimum inhibitory concentrations of genistein and daidzein to be 400 µM and 500 µM, respectively^3^. We discovered that in the presence of 400 µM genistein in vitro, R5 degraded genistein and produced indole alkaloids^21^. To assess whether R5 responds to daidzein similarly, we performed a comparative metabolomic analysis on cell cultures. We lowered the concentration of daidzein to that of genistein rather than use any additional genistein. Thus, R5 liquid cultures were separately exposed to 400 µM genistein, 400 µM daidzein, or an equivalent volume of control dimethyl sulfoxide (DMSO), and the cell extracts were analyzed by non-targeted mass spectrometry. There were 1,719 compound features found in positive ion mode and 2,072 found in negative ion mode. The main compounds from genistein-treated cells with significantly increased accumulation (calculated from compound chromatographic peak areas) were genistein and its degradation products of smaller mass like 2,3-dihydro-4 H-chrome-4-one or its converted products like 5-O-methylgenistein with greater mass (Fig. 1; Supplementary Dataset S1). The main compounds from daidzein-treated cells with significantly increased accumulation were daidzein and its degradation products like dioxybenzone or its converted products like formononetin (Fig. 1; Supplementary Dataset S1). These compounds were identified by tandem mass spectrum spectral library matching for Level 2 identification^25,26^ (Supplementary Dataset S1). In addition to accumulating these specific degradation products and derivatives of daidzein and genistein, the cells had increased amounts of indole compounds such as N-acetylserotonin and trans-3-indoleacrylic acid (Fig. 1; Supplementary Dataset S1). Some indoles such as 4-indolecarbaldehyde, indole-3-propionitrile, 2-(2-piperidinyl)-1 H-indole, and an isomer of indole-3-acetic acid, as well as several indole beta-carbolines like harmaline, harmol, harmane, and norharman that were previously found in genistein-treated cells^21^ were induced to a higher extent in daidzein-treated cells (Fig. 1; Supplementary Dataset S1). Daidzein- and genistein-treated *P. savastanoi* pv. *phaseolicola* R5 also produced phenylacetoacetonitrile, a derivative of phenylacetonitrile and phenylacetic acid that each have indole-3-acetic acid-like plant hormonal activity (Fig. 1; Supplementary Dataset S1)^27,28^. Overall, these findings demonstrate that *P. savastanoi* pv. *phaseolicola* R5 catabolizes daidzein and genistein and responds by producing indole compounds. We hypothesize that some of the indole compounds could favor bacterial pathogenicity, possibly by modifiying plant host metabolism through auxin-like hormonal pathways in a way that indole-3-acetic acid (auxin) does for *P. syringae*^29^.

Another class of compounds induced by daidzein and genistein was cyclic dipeptides. Four of the notable cyclic dipeptides were formed with proline, namely cyclo-Phe-Pro (cFP), cyclo-Gly-Pro (cGP), cyclo-Leu-Pro (cLP), and cyclo-Trp-Pro (cWP) (Fig. 1; Supplementary Dataset S1). Cyclic dipeptides are intriguing because some like cFP, cLP, and cyclo-Tyr-Pro function in bacterial quorum sensing^30^ while others like cFP, cyclo-Val-Pro, and cyclo-Tyr-Pro may promote auxin-like cell growth in *A. thaliana*^31^. Thus, we hypothesize that the bacterial production of cyclic dipeptides in response to genistein or daidzein could support pathogenicity.

### Cyclic dipeptides alter small molecule accumulation in bean leaves

**Fig. 2:**
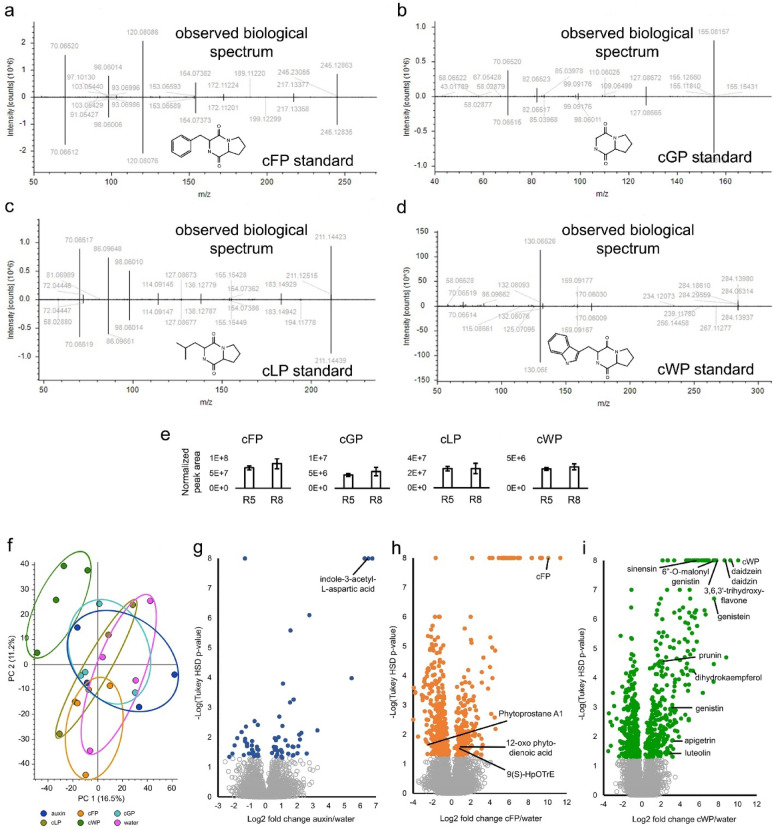
Cyclic dipeptides from *P. savastanoi* pv. *phaseolicola*, and metabolomics after *P. vulgaris* infiltration. (**a**–**d**) Mirror plots of tandem mass spectra for cyclic dipeptides for (**a**) cFP, (**b**) cGP, (**c**) cLP, and (**d**) cWP produced in genistein- and daidzein-treated *P. savastanoi* pv. *phaseolicola* R5 versus chemical standards. The observed spectrum is on top of each plot and the standard is on the bottom. (**e**) Mean chromatographic peak areas of cFP, cGP, cLP, and cWP in daidzein-treated *P. savastanoi* pv. *phaseolicola* R5 and R8 [error bars depict standard deviation (the differences are not significant); *n* = 5]. (**f**) Principal component (PC) analysis of compounds detected by mass spectrometry in positive ion mode from *P. vulgaris* infiltrated with 30 µM auxin, cFP, cGP, cLP, cWP, or water 24 h after infiltration (*n* = 4). (**g**–**i**) Volcano plots of fold changes of compounds from *P. vulgaris* treated with (**g**) 30 µM auxin, (**h**) 30 µM cFP, and (**i**) 30 µM cWP versus water-treated controls as a function of − log10(*p*-value) of their chromatographic peak areas. For (g-i), compounds with FDR < 5% (Tukey HSD p-value < 1%) and significant fold-changes are represented as solid color dots, and those without significant fold-changes or with FDR > 5% are represented as gray circles. cFP, cyclo-Phe-Pro; cGP, cyclo-Gly-Pro; cLP, cyclo-Leu-Pro; and cWP, cyclo-Trp-Pro.

To begin assessing any potential roles these bacterial compounds may have as pathogenicity factors, we examined whether they could alter plant cell metabolism to favor bacterial infection. We focused on the cyclic dipeptides because cFP, cyclo-Val-Pro, and cyclo-Tyr-Pro promote weak, auxin-like cell growth in *A. thaliana*^31^. We first confirmed the identification of the cyclic dipeptides using pure standards (Fig. 2a–d). We also demonstrated that *P. savastanoi* pv. *phaseolicola* race 8 (R8) produces similar amounts of the same cyclic dipeptides as R5 when treated with 400 µM daidzein (Fig. 2e). R5 elicits hypersensitive immunity on Black Valentine beans whereas R8 does not^24^. Thus, cFP, cGP, cLP, and cWP are made by virulent and avirulent *P. savastanoi* pv. *phaseolicola* races.

Next, we infiltrated 30 µM concentrations of cFP, cGP, cLP, and cWP into bean leaves. We chose these concentrations because cFP induced auxin-like growth activity in *A. thaliana* at the same concentration^31^. Speculating these cyclic dipeptides may incite auxin-like activity in beans, especially cWP which has an indole functional group, we tested 30 µM indole-3-acetic acid (auxin) for comparison. Principle Component analysis of 4,274 positive ion mode compound features resolved by mass spectrometry performed on infiltrated leaf extracts demonstrated that only cWP-treatment compound features clustered separately from the others (Fig. 2f). Statistical analysis of compound peak areas revealed that auxin treatment led to the fewest significant changes in abundance (67), the main identifiable compound being a conjugated, inactivated form of indole-3-acetic acid (Fig. 2g; Supplementary Dataset S2). cLP treatment produced 171 significant changes in compound abundance whereas cGP produced 207 (Supplementary Dataset S2). The infiltrated cLP and cGP were the main compounds identified in each case, but there were few other identifiable compounds that we could link to a plausible metabolic response model. cFP treatment led to 543 significant changes including significant increases in 12-oxo phytodienoic acid and 9(S)-HpOTrE, each of the alpha-linolenic acid pathway (Fig. 2h; Supplementary Dataset S2). Phytoprostane A1, also part of the alpha-linolenic acid pathway, decreased (Fig. 2h; Supplementary Dataset S2). These findings imply that cFP may have affected parts of fatty acid biosynthesis, especially the alpha-linolenic acid pathway that leads to jasmonic acid biosynthesis. Prior research has linked cFP to alterations in auxin-like hormonal growth responses in *A. thaliana*^31^, but we did not observe responses with cFP consistent with auxin treatment. Consequently, the results for cFP are difficult to interpret regarding pathology and may warrant deeper investigations in beans.

Compared to these cyclic dipeptides and auxin, cWP induced the most compound abundance changes (686; Supplementary Dataset S2). Amongst them were increases in daidzein and genistein and their glycosylated derivatives daidzin, genistin, and 6”-O-malonylgenistin (Fig. 2i). Several structurally similar flavonoids and glycosylated isoflavonoids also were induced (Fig. 2i; Supplementary Dataset S2). We repeated bean leaf infiltrations with 30 µM cWP. Again, we observed drastic increases in daidzein, genistein and their derivatives, and greater increases in other flavonoids and isoflavonoids including daidzein-derived coumestrol (Supplementary Dataset S3). These findings reveal that cWP, but not cFP, cGP, or cLP, elicits phytoalexin production in beans.

### cWP induces disease resistance in beans

**Fig. 3:**
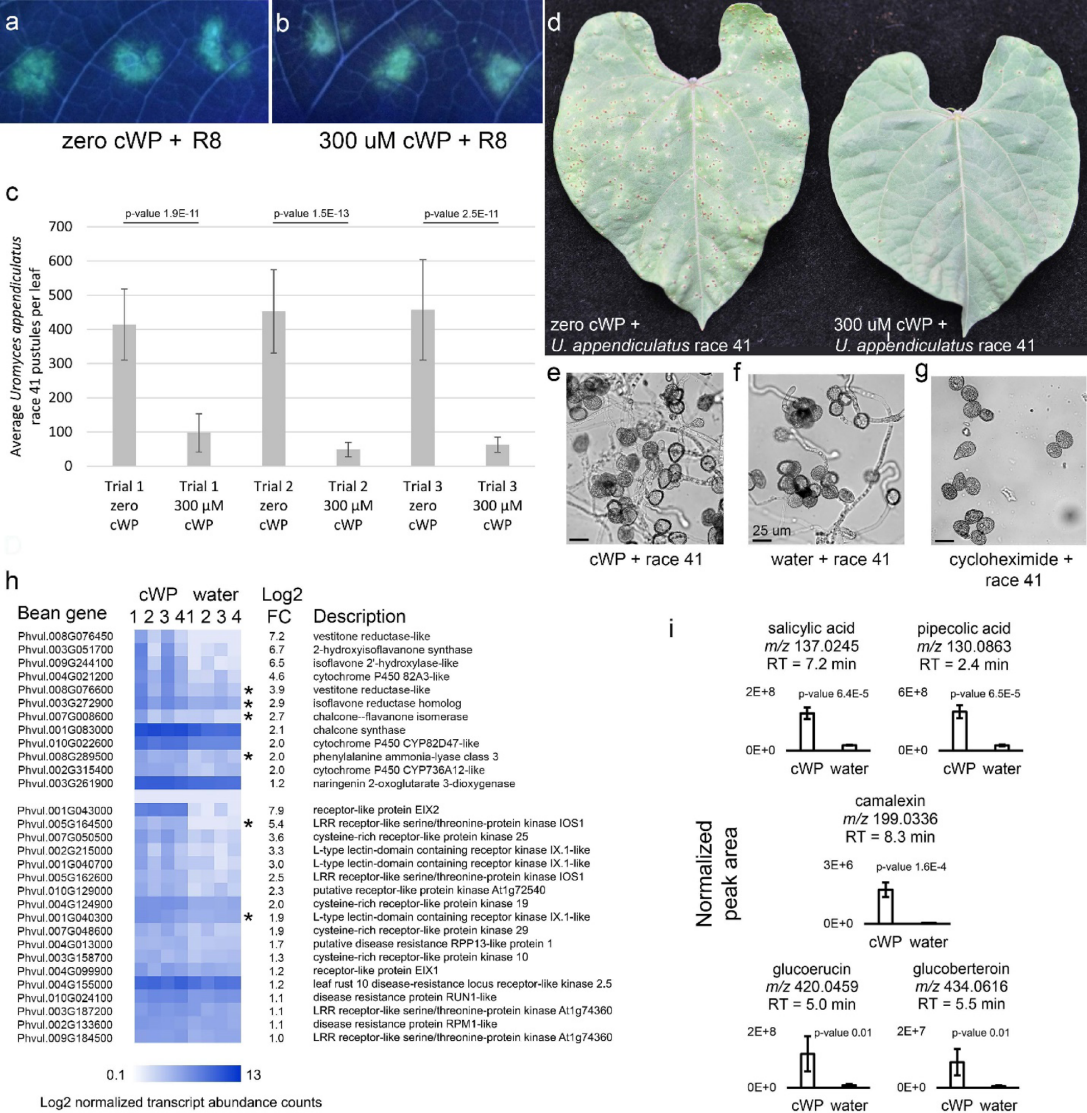
Effects of cWP on *P. vulgaris* variety Black Valentine, *U. appendiculatus* (rust) spores, and *A. thaliana.* (**a**) Autofluorescence (transillumination 366 nm) of *P. savastanoi* pv. *phaseolicola* race 8 (R8) 4 days after inoculation on Black Valentine bean leaves sprayed with water 24 h prior to inoculation. (**b**) Autofluorescence (transillumination 366 nm) of R8 4 days after inoculation on Black Valentine sprayed with 300 µM cWP 24 h prior to inoculation. [There is no perceptible difference in spread of R8 between (**a**) and (**b**)]. (**c**) Average number of *U. appendiculatus* race 41 pustules on water-treated or 300 µM cWP-treated Black Valentine. Leaves were inoculated 24 h after cWP or water treatment. Three trials were performed (p-values are for t-test comparisons of numbers of pustules; error bars represent standard deviation; *n* = 16). (**d**) Signs of *U. appendiculatus* race 41 disease (rust pustules) on primary leaves of Black Valentine treated with water or 300 µM cWP. (**e**) *U. appendiculatus* race 41 spore germlings on a glass slide sprayed with 300 µM cWP. (**f**) *U. appendiculatus* race 41 spore germlings on a glass slide sprayed with water. (**g**) Lack of *U. appendiculatus* race 41 spore germlings on a glass slide sprayed with 5% cycloheximide. (**h**) Statistically significant Log2 normalized transcript abundance counts of genes in Black Valentine leaves 24 h after 300 µM cWP or water treatment. FC is fold-change for cWP vs. water (*n* = 4). Genes with corresponding proteins induced by benzothiadiazole treatment of Black Valentine leaves are marked with an asterisk. (**i**) Mean chromatographic peak areas of compounds found by tandem mass spectrometry in *A. thaliana* treated with 300 µM cWP or water (p-values are for t-test comparisons of normalized peak areas; error bars represent standard deviation; *n* = 6).

We tested whether phytoalexin increases induced by cWP treatment affected disease resistance. Experimental challenges with virulent *P. savastanoi* pv. *phaseolicola* R8 on bean leaves pretreated 24 h in advance with cWP revealed no reduction in bacterial spread and no delay in appearance of bacteria compared to water-pretreated controls as visualized by bacterial autofluorescence (Fig. 3a,b). We speculated that R8 might be adapted to overcoming the phytoalexin response initiated by cWP. So instead, we tested *Uromyces appendiculatus* race 41, a rust fungus virulent on *P. vulgaris* variety Black Valentine. The asexual rust uredospores applied to the surface of the leaf will germinate in dew, and their hyphae will actively enter the leaf apoplast through leaf stomata based on thigmotropic recognition^32^. The number of resulting leaf pustules, the asexual fruiting bodies, is directly proportional to the number of successful fungal infections. Preliminary tests with rust on beans treated with 30 µM cWP revealed a potential 50% reduction in pustules. When we increased the concentration of cWP to 300 µM, we observed 75–90% reductions in numbers of pustules when cWP was applied to primary bean leaves 24 h prior to inoculation with rust spores (Fig. 3c,d). To see if cWP was directly inhibiting rust spore germination, we sprayed glass microscope slides with 300 µM cWP and then sprayed rust spores on the slides as we would for leaf inoculation. After overnight incubation in a dew chamber, spores exposed to cWP germinated normally and had elongated hyphae that appeared to be the same as water-treated slide controls (Fig. 3e,f). By contrast, there was near-complete germination inhibition on slides sprayed with 5% cycloheximide, a natural fungicide produced by *Streptomyces griseus* (Fig. 3g). These results reveal that cWP is not fungicidal, per se, in its efficacy toward *U. appendiculatus* but rather functions by increasing the amounts of phytoalexins in leaves.

RNA sequencing of cWP-treated beans confirmed increased expression of genes for enzymes that catalyze key steps of isoflavonoid biosynthesis including phenylalanine ammonia lyase, chalcone synthase, and 2-hydroxyisoflavonone synthase genes (Fig. 3h; Supplementary Dataset S4). Furthermore, there was increased expression of leucine-rich repeat and cysteine-rich receptor kinases and other candidate immune receptors and signaling proteins (Fig. 3h; Supplementary Dataset S4). A few of these genes had increased abundances of proteins in beans sprayed with benzothiadiazole, an unrelated synthetic chemical that induces immunity to rust^33^ (Fig. 3h; Supplementary Dataset S4). These results revealed that not only did cWP induce phytoalexin production, but it also primed the bean immune system for the detection of pathogens and for concomitant immune system signaling. This attribute also was observed in cWP-sprayed *A. thaliana* which subsequently accumulated six times more salicylic acid and pipecolic acid, regulators of acquired and systemic acquired resistance^34,35^ (Fig. 3i; Supplementary Dataset S5). Camalexin, a tryptophan-derived phytoalexin controlled by the salicylic acid defense pathway^36^, increased by 32-fold, and bactericidal glucosinolates glucoerucin and glucoberteroin increased by more than 15-fold (Fig. 3i; Supplementary Dataset S5)^37^.

## Discussion

Over the last few decades we have learned exquisite details about the interactions between plants and their pathogens and how this leads to disease susceptibility or immunity^38^. The immune response includes physiological, transcriptional, translational, post-translational, enzymatic, and metabolic responses. Specific metabolic responses include the production of phytoalexins, small molecules with antibiotic potential that accumulate at the site of infection to stop disease progression. The accumulation of a variety of phytoalexins and other toxic compounds could explain why induced immunity kills most invading bacteria^39^, although additional mechanisms of immunity could lead to the starvation, dehydration, or immobilization of bacterial invaders^39–42^. Nevertheless, how plant immunity output, including phytoalexin toxicity, acts upon bacterial pathogens and how bacterial pathogens respond to plant immune system output remain among some of the crucial but understudied topics in the field.

We have reopened investigation into these topics and demonstrate here that *P. savastanoi* pv. *phaseolicola*, a bacterial pathogen of beans, metabolically degrades genistein and daidzein, the two major isoflavonoid phytoalexins of beans, and responds by producing indoles and cyclic dipeptides (Fig. 1). These indoles and cyclic dipeptides contain nitrogen whereas genistein and daidzein do not. Thus, they are produced in response to, not from, genistein and daidzein. These findings reveal an intriguing possiblity whereby this adapted pathogen of beans perceives genistein and daidzein, isoflavonoids that differ by a hydroxylation moiety^10^, as a signals to drive infection. This is consistent with observations that symbiotic rhizobia of soybean and pathogenic *P. sojae* tolerate genistein and daidzein^23,43^, and that genistein and daidzein exudates from roots serve as signals to direct rhizobia symbiosis and *P. sojae* infections in soybean, a legume related to bean^22^.

These observations prompted us to investigate whether the induced bacterial compounds assisted infection by modifying plant cell metabolism. In particular, we focussed on the potential biological activity of the cyclic dipeptides. While it is known that bacteria use cyclic dipeptides like cLP, cFP, cyclo-Tyr-Pro, and cyclo-Val-Pro for bacterial quorum sensing^30,31,44^, only a few studies have shown the effects of cyclic dipeptides on plants. In *A. thaliana*, cFP, cyclo-Tyr-Pro and cyclo-Val-Pro induced weak auxin-like effects^31^. In *Nicotiana benthamiana*, cyclo-Pro-Pro (but not cFP) induced local acquired resistance to *Phytophthora nicotianae*^45^. Our metabolomic investigation revealed that neither cFP, cGP, cLP, nor cWP elicited a metabolic response similar to a herbicidal concentration of indole-3-acetic acid (Fig. 2g–i). Rather, we found that cWP elicited phytoalexin production in beans and primed immune system gene expression (Figs. 2i and 3h). These changes likely contributed to increased resistance to rust fungal infection (Fig. 3c,d). Because cWP also elicited phytoalexin and glucosinolate production in *A. thaliana* (Fig. 3i), it is possible that other plants will respond to cWP in a similar way, and that cWP, a natural compound, can be used to deter multiple diseases in numerous crops.

### Limitations and future directions

Whether cyclic dipeptides assist *P. savastanoi* pv. *phaseolicola* quorum sensing or are produced to serve other bacterial functions remains to be determined. Nevetheless, our discovery of the bacterial production of cWP upon treatment with plant phytoalexins and the induction of phytoalexins by cWP implies there is a hypothetical biochemical feedback loop between the host and its pathogen. Although *P. savastanoi* pv. *phaseolicola* encounters amplified amounts of genistein and daidzein during an immune reaction in beans^3^ and increases its cWP production in the presence of an inhibitory amount of genistein and daidzein in vitro (Fig. 1), we do not know if bacterial cWP is made at the site of infection or if bacterial cWP induces bean immune signaling during infection. A recent report revealing that *A. thaliana* produces cyclo-His-Pro during abiotic stress responses opens the door to the possibility that beans could also make cWP or other cyclic dipeptides^46^. If so, does this mean that plant cWP is regulated by the plant immune system? Is there a cWP plant receptor that can recognize bacterial cWP? Does the amount of bacterial cWP overcome a plant cWP threshold to induce biochemical immunity feedback? These intriguing questions deserve future attention.

## Methods

### Bacterial strains and genistein and daidzein treatment

*P. savastanoi* pv. *phaseolicola* isolates race 5 (R5) and 8 (R8) were maintained on King Agar B solid medium (Sigma-Aldrich, St. Louis, MO) supplemented with glycerol. The genome sequences for R5 and R8 are reported^47^. Genistein and daidzein were obtained from Sigma-Aldrich and were solubilized in dimethyl sulfoxide (DMSO). R5 and R8 were cultured in 10 mL Luria broth with 400 µM genistein, 400 µM daidzein, or without but with 50 µL DMSO at 28 °C at 200 rpm on a shaking platform until the control cultures reached late log-phase growth measured at an optical density of 0.75–0.90 at 600 nm wavelength of visible light.

### Mass spectrometry of metabolites

Five replicate R5 cultures of each treatment were centrifuged at 5,000 x *g* for 10 min, washed once in 1 mL phosphate buffered saline, centrifuged at 5,000 x *g* for 10 min, resuspended in 1 mL of 1:1:1:1 acetone/acetonitrile/methanol/water and 1,250 pmol prednisone, and pulverized with 0.5 mm glass beads with a Qiagen PowerLyzer 24 bead mill (Qiagen, Hilden, Germany) 10 times at 5,000 rpm for 20 s (cooled on ice for 2 min between cycles). Two matrix blank samples were prepared the same way starting with 10 mL Luria broth (without R5 and without genistein or daidzein). The milled extracts were centrifuged for 20 min at 12,000 x *g.* The supernatants were transferred to fresh tubes and centrifuged again. The supernatants were transferred to glass vials and dried under vacuum. The residues were resuspended in 115 µL 50% methanol/0.1% formic acid. Fifteen µL of each sample was pooled to create a quality control (QC) sample. Five µL of the samples were separated on a 150 × 2.1 mm Hypersil GOLD VANQUISH HPLC column with 1.9 μm particles (Thermo Fisher Scientific) coupled to a Vanquish HPLC pump (Thermo Fisher Scientific) controlling a 10-min linear gradient from 0% to 95% acetonitrile and 0.1% formic acid at a flow rate of 0.2 mL per min. Eluent was electrosprayed at 3.5 kV positive polarity into an Exploris 240 mass spectrometer (Thermo Fisher Scientific) using an internal mass calibrant. Sheath gas was 35, auxiliary gas was 7, and sweep gas was 1 (arbitrary units). The ion transfer tube temperature was 325 °C, and the vaporizer temperature was 275 °C. Advanced peak determination, mild trapping, and internal mass calibration was enabled. Default charge state was 1, and the expected peak width was 6 s. AcquireX software was used to create a background ion exclusion list from the matrix blank sample and an inclusion ion list from the QC sample^48^. Five subsequent injections of the QC were performed to generate tandem mass spectra (MS^2^). After each QC injection, the resolved ions were appended to the exclusion list. Survey scans were recorded in the Orbitrap at 60,000 resolution over a range of *m/z* 70–800. The RF lens was 70%. Monoisotopic precursor selection was enabled, the minimum intensity was 5,000, and charge states were filtered to 1. Precursors selected within a 1.0 Da isolation window were fragmented by high energy collision-induced dissociation (30%, 50%, 70% normalized stepped collision energy), and fragment ions were resolved in the Orbitrap at 30,000 resolution. Subsequently, all test samples were analyzed alongside QC and matrix blank samples in the following order: Matrix blank 1, QC 1, sample replicate set 1, Blank 2, QC 2, sample replicate set 2, etc. Survey scans were recorded in the Orbitrap at 120,000 resolution over a range of *m/z* 70–800. The RF lens was 70%. The samples also were analyzed in negative ion mode at -2,500 V but with the same other settings.

Result raw files were analyzed with Compound Discoverer version 3.3 (Thermo Fisher Scientific). The ChromAlign node was used to align chromatographic peaks in all files to a QC replicate. The Detect Compounds node was used with 2 ppm mass tolerance, 10,000 minimum peak intensity, at least 5 scans per peak, 0.25 min maximum peak width, and compound detection of [M + H] + 1 ions for positive mode data and [M-H]-1 ions for negative mode data to create peak areas for resolved ions. The Group Compounds node was used with 2 ppm mass tolerance, 0.25 min retention time (RT) tolerance, and a peak rating threshold of 4 for a minimum of 6 files. The Fill Gaps node was used with 2 ppm mass tolerance, the SERRF QC Correction node was used with 80% QC coverage to normalize the peak area results, and the Mark Background Components node was enabled. The Search mzCloud node was used to compare MS^2^ spectra to all compound classes at a precursor mass tolerance of 5 ppm and fragment mass tolerance of 5 ppm. The Search mzVault node was used to compare MS^2^ spectra to the NIST_2020_MSMS High Resolution library at fragment mass tolerances of 10 ppm. The Assign Compound Annotations, Map to KEGG Pathways, Search ChemSpider, and Predict Compositions nodes were used with 2 ppm mass tolerances. A one-way ANOVA with a Tukey post-hoc test was used to estimate compound peak area statistical differences. The Benjamini-Hochberg method was used to adjust the p-values. Filtering of results was performed to limit background ions, to require MS^2^ supporting spectra, for the false discovery rate (FDR) to be less than 5%, and mzCloud or NIST2020_MSMS scores ≥ 70 (Supplementary Dataset S1).

### Cyclic dipeptides and application to bean and *Arabidopsis thaliana* leaves

Cyclo-L-phenylalanine-L-proline (cFP) was obtained from Toronto Research Chemicals (Toronto, ON, Canada). Cyclo-L-leucine-L-proline (cLP) and cyclo-L-tryptophan-L-proline (cWP) were obtained from Ambeed (Arlington Hts, IL). Cyclo-glycine-L-proline (cGP) was obtained from Accela (San Diego, CA). Approximately 1 nmol of each cyclic dipeptide was analyzed for RT on the reverse phase column and to produce a reference tandem mass spectrum under the mass spectrometry conditions noted above.

Primary leaves of 10-day-old *P. vulgaris* (common bean) variety Black Valentine plants were infiltrated with 30 µM cFP, cGP, cLP, cWP, or auxin (indole-3-acetic acid, Sigma Aldrich), or water (control) using a flat-nosed 1 mL syringe. We chose these concentrations because cFP induced auxin-like growth activity in *A. thaliana* at the same concentration^31^. The two primary leaves on each plant were each infiltrated 8 times (4 times on each half leaf) with a single compound. Infiltration was performed on the abaxial side without puncturing the leaf, and each infiltration produced a 1 cm^2^ water-soaked area. After 24 h, the leaf tissue at the site of infiltration was sampled with a 1 cm diameter cork-borer. Ten to twelve sites were collected from each plant to reach approximately 170 mg total tissue which was extracted in 1 mL 100% methanol using glass beads as described above. Four separate plants of each treatment were used to generate four replicates. A one-way ANOVA with a Tukey post-hoc test was used to estimate compound peak area statistical differences (Supplementary Dataset S2). In a later, focused experiment to compare cWP to water, seven replicates were used, and the tissues were collected at 24 h after infiltration. Non-targeted mass spectrometry was performed the same as above, but reference library searches included the cyclic dipeptide reference spectra. An empirical Bayes statistics test was used in the LIMMA package in the R programming environment to assess compound normalized peak area statistical differences and to estimate the FDR (the Benjamini-Hochberg method). Filtering of results was performed to limit background ions, to require MS^2^ supporting spectra, for the FDR to be less than 5%, and mzCloud or NIST2020_MSMS scores ≥ 70 (Supplementary Dataset S3).

R8 was cultured on King Agar B solid medium supplemented with glycerol, resuspended in water, and adjusted to an optical density at 600 nm = 2. The abaxial sides of two primary leaves of 10-day-old *P. vulgaris* variety Black Valentine were each inoculated 10 times using a Master airbrush at 50 psi at a 1- to 2-cm distance. Water was sprayed as a control. Bacterial infection and spread was assessed by bacterial autofluorescence visualized by transillumination at 366 nm.

Two primary leaves from 8, 10-day-old *P. vulgaris* variety Black Valentine plants were sprayed with a liquid suspension of 300 µM cWP or water. A day later, the leaves were inoculated with a liquid suspension of uredospores of *U. appendiculatus* race 41 and placed in an 18 °C dew chamber for 12 h and then moved to a 23 °C growth chamber with fluorescent lighting and 50% relative humidity^49^. Inoculum was adjusted to produce 400–500 pustules per leaf. Pustules were enumerated on all plants when they appeared on water-treated controls inoculated with race 41. Sixteen leaves were examined per treatment, and three biological replicate experiments were evaluated. A two-tailed Student’s t-test was used to assess differences in numbers of pustules.

To assess spore germination, glass slides were sprayed with 300 µM cWP, water, or 5% cycloheximide (germination inhibition positive control). When the slides dried, they were sprayed with a liquid suspension of uredospores of *U. appendiculatus* race 41 and placed in an 18 °C dew chamber. Spore germination was assessed by light microscopy 18 h later.

Primary leaves from four Black Valentine plants were sprayed with a liquid suspension of 300 µM cWP or water. 24 h later, RNA was purified from the leaves using the Qiagen RNeasy Plant Mini Kit. mRNA sequencing via polyA selection was performed by Genewiz (South Plainfield, NJ) with an Illumina 2 × 150 bp platform. The overall project yielded 164 × 10^6^ reads, and 49 × 10^3^ Mbases with a mean quality score of 38.02. Sequence reads were trimmed to remove adapter sequences and nucleotides with poor quality using Trimmomatic v.0.36. The trimmed reads were mapped to the Phaseolus_vulgaris_2_0 reference genome available on ENSEMBL using STAR aligner v.2.5.2b. Unique gene hit counts were calculated by using featureCounts from the Subread package v.1.5.2. The hit counts were summarized and reported using the gene_id feature in the annotation file. Only unique reads that fell within exon regions were counted. Comparisons of gene expression between water and cWP-treated groups of samples were performed using DESeq2. The Wald test was used to generate p-values and log2 fold changes. Genes (1,341) with an adjusted p-value < 0.05 and absolute log2 fold change > 1 were called as differentially expressed genes. Gene names were linked to corresponding JGI accession numbers, *A. thaliana* gene orthologs, Gene Ontology process and function terms, and differentially accumulating proteins from benzothiadiazole-treated Black Valentine^33^ (Supplementary Dataset S4).

Leaves of 3-week-old *A. thaliana* ecotype Columbia-0 were sprayed with 300 µM cWP or water. After 24 h, approximately 150 mg of leaf tissue was collected from 7 to 10 plants and pooled to form a replicate. This was performed six times for each treatment. The samples were prepared and analyzed by mass spectrometry as described above. A two-tailed Student’s t-test was used to assess compound normalized peak area statistical differences and p-values were adjusted by the Benjamini-Hochberg method (Supplementary Dataset S5).

## Supplementary Information

Below is the link to the electronic supplementary material.

Supplementary Material 1

Supplementary Material 2

Supplementary Material 3

Supplementary Material 4

Supplementary Material 5

## Data Availability

Mass spectrometry data files can be retrieved from https://massive.ucsd.edu/. (1) Pseudomonas savastanoi pv. phaseolicola race 5 treated with genistein, daidzein, and DMSO. (MSV000096150). (2) Phaseolus vulgaris infiltrated with cyclic dipeptides and auxin. (MSV000096164). (3) Phaseolus vulgaris infiltrated with cWP and water. (MSV000096165). (4) Arabidopsis thaliana sprayed with cWP or water. (MSV000096166).

## Abbreviations

DMSO: Dimethyl sulfoxide
QC: Quality control
MS^2^: Tandem mass spectra
RT: Retention time
FDR: False discovery rate
cFP: Cyclo-L-phenylalanine-L-proline
cLP: Cyclo-L-leucine-L-proline
cWP: Cyclo-L-tryptophan-L-proline
cGP: Cyclo-glycine-L-proline
PC: Principal component

## Declarations

### Permits and permissions

*Pseudomonas savastanoi* pv. *phaseolicola* and *Uromyces appendiculatus* are authorized for use under controlled conditions by permit from USDA-APHIS. All infected plant materials were destroyed after experimentation. Relevant agency and national guidelines are outlined by USDA-ARS research project 8042-21220-261-000-D.

## References

[1] Piasecka, A., Jedrzejczak-Rey, N. & Bednarek, P. Secondary metabolites in plant innate immunity: conserved function of divergent chemicals. *New Phytol.* **206**, 948–964 (2015). 25659829 10.1111/nph.13325

[2] Kuc, J. Phytoalexins, stress metabolism, and disease resistance in plants. *Annu. Rev. Phytopathol.* **33**, 275–297 (1995). 18999962 10.1146/annurev.py.33.090195.001423

[3] Cooper, B., Campbell, K. B. & Garrett, W. M. Salicylic acid and phytoalexin induction by a bacterium that causes halo blight in beans. *Phytopathology* **112**, 1766–1775 (2022). 35147446 10.1094/PHYTO-12-21-0496-R

[4] Thomma, B. P. H. J., Nelissen, I., Eggermont, K. & Broekaert, W. F. Deficiency in phytoalexin production causes enhanced susceptibility of Arabidopsis Thaliana to the fungus alternaria brassicicola. *Plant J.* **19**, 163–171 (1999). 10476063 10.1046/j.1365-313x.1999.00513.x

[5] Glazebrook, J. & Ausubel, F. M. Isolation of phytoalexin-deficient mutants of Arabidopsis Thaliana and characterization of their interactions with bacterial pathogens. *Proc. Natl. Acad. Sci. U S A*. **91**, 8955–8959 (1994). 8090752 10.1073/pnas.91.19.8955 PMC44725

[6] Graham, T. L., Graham, M. Y., Subramanian, S. & Yu, O. RNAi Silencing of genes for elicitation or biosynthesis of 5-deoxyisoflavonoids suppresses race-specific resistance and hypersensitive cell death in phytophthora Sojae infected tissues. *Plant. Physiol.* **144**, 728–740 (2007). 17416637 10.1104/pp.107.097865 PMC1914209

[7] Hain, R. et al. Disease resistance results from foreign phytoalexin expression in a novel plant. *Nature* **361**, 153–156 (1993). 8421520 10.1038/361153a0

[8] He, X. Z. & Dixon, R. A. Genetic manipulation of isoflavone 7-O-methyltransferase enhances biosynthesis of 4’-O-methylated isoflavonoid phytoalexins and disease resistance in alfalfa. *Plant. Cell.* **12**, 1689–1702 (2000). 11006341 10.1105/tpc.12.9.1689 PMC149079

[9] Ahuja, I., Kissen, R. & Bones, A. M. Phytoalexins in defense against pathogens. *Trends Plant. Sci.* **17**, 73–90 (2012). 22209038 10.1016/j.tplants.2011.11.002

[10] Kim, M., Han, J. & Kim, S. U. Isoflavone daidzein: chemistry and bacterial metabolism. *J. Appl. Biol. Chem.* **51**, 253–261 (2008).

[11] Ogawara, H., Akiyama, T., Ishida, J., Watanabe, S. & Suzuki, K. A specific inhibitor for tyrosine protein kinase from Pseudomonas. *J. Antibiot.* **39**, 606–608 (1986). 10.7164/antibiotics.39.606 3710920

[12] Akiyama, T. et al. Genistein, a specific inhibitor of tyrosine-specific protein kinases. *J. Biol. Chem.* **262**, 5592–5595 (1987). 3106339

[13] Xagorari, A. et al. Luteolin inhibits an endotoxin-stimulated phosphorylation cascade and Proinflammatory cytokine production in macrophages. *J. Pharmacol. Exp. Ther.* **296**, 181–187 (2001). 11123379

[14] Nakashima, S., Koike, T. & Nozawa, Y. Genistein, a protein tyrosine kinase inhibitor, inhibits thromboxane A2-mediated human platelet responses. *Mol. Pharmacol.* **39**, 475–480 (1991). 2017148

[15] Hong, H., Landauer, M. R., Foriska, M. A. & Ledney, G. D. Antibacterial activity of the soy isoflavone genistein. *J. Basic Microbiol.* **46**, 329–335 (2006). 16847837 10.1002/jobm.200510073

[16] Wells, C. L., Jechorek, R. P., Kinneberg, K. M., Debol, S. M. & Erlandsen, S. L. The isoflavone genistein inhibits internalization of enteric bacteria by cultured Caco-2 and HT-29 enterocytes. *J. Nutr.* **129**, 634–640 (1999). 10082767 10.1093/jn/129.3.634

[17] Verdrengh, M., Collins, L. V., Bergin, P. & Tarkowski, A. Phytoestrogen genistein as an anti-staphylococcal agent. *Microbes Infect.* **6**, 86–92 (2004). 14738897 10.1016/j.micinf.2003.10.005

[18] Cooper, B. et al. Quantitative proteomic analysis of Staphylococcus aureus treated with Punicalagin, a natural antibiotic from pomegranate that disrupts iron homeostasis and induces SOS. *Proteomics* **18**, e1700461 (2018). 29528570 10.1002/pmic.201700461

[19] Cooper, B. The detriment of Salicylic acid to the Pseudomonas Savastanoi pv. phaseolicola proteome. *Mol. Plant. Microbe Interact.* **35**, 814–824 (2022). 35612310 10.1094/MPMI-05-22-0104-R

[20] Cooper, B. Disruptive effects of Resveratrol on a bacterial pathogen of beans. *J. Proteome Res.* **22**, 204–214 (2023). 36512343 10.1021/acs.jproteome.2c00633

[21] Cooper, B., Yang, R. & Campbell, K. B. Indole alkaloid production by the halo blight bacterium treated with the phytoalexin genistein. *Phytopathology* **114**, 1196–1205 (2024). 38281161 10.1094/PHYTO-11-23-0445-R

[22] Kosslak, R. M., Bookland, R., Barkei, J., Paaren, H. E. & Appelbaum, E. R. Induction of Bradyrhizobium Japonicum common Nod genes by isoflavones isolated from Glycine max. *Proc. Natl. Acad. Sci. U S A*. **84**, 7428–7432 (1987). 16593884 10.1073/pnas.84.21.7428 PMC299309

[23] Morris, P. F., Bone, E. & Tyler, B. M. Chemotropic and contact responses of phytophthora Sojae hyphae to soybean isoflavonoids and artificial substrates. *Plant. Physiol.* **117**, 1171–1178 (1998). 9701573 10.1104/pp.117.4.1171 PMC34881

[24] Cooper, B., Campbell, K. B., Beard, H. S., Garrett, W. M. & Ferreira, M. E. The proteomics of resistance to halo blight in common bean. *Mol. Plant. Microbe Interact.* **33**, 1161–1175 (2020). 32633604 10.1094/MPMI-05-20-0112-R

[25] Schymanski, E. L. et al. Identifying small molecules via high resolution mass spectrometry: communicating confidence. *Environ. Sci. Technol.* **48**, 2097–2098 (2014). 24476540 10.1021/es5002105

[26] Sumner, L. W. et al. Proposed minimum reporting standards for chemical analysis chemical analysis working group (CAWG) metabolomics standards initiative (MSI). *Metabolomics: Official J. Metabolomic Soc.* **3**, 211–221 (2007). 10.1007/s11306-007-0082-2 PMC3772505 24039616

[27] Wheeler, A. W. Auxin-Like growth activity of phenylacetonitrile. *Ann. Bot-London*. **41**, 867–872 (1977).

[28] Perez, V. C., Zhao, H., Lin, M., Kim, J. & Occurrence function, and biosynthesis of the natural auxin phenylacetic acid (PAA) in plants. *Plants (Basel)* **12** (2023). 10.3390/plants12020266 PMC9867223 36678978

[29] Djami-Tchatchou, A. T. et al. Dual role of auxin in regulating plant defense and bacterial virulence gene expression during Pseudomonas syringae PtoDC3000 pathogenesis. *Mol. plant-microbe Interactions: MPMI*. **33**, 1059–1071 (2020). 32407150 10.1094/MPMI-02-20-0047-R PMC7810136

[30] Degrassi, G. et al. Plant growth-promoting Pseudomonas Putida WCS358 produces and secretes four Cyclic dipeptides: cross-talk with quorum sensing bacterial sensors. *Curr. Microbiol.* **45**, 250–254 (2002). 12192521 10.1007/s00284-002-3704-y

[31] Ortiz-Castro, R. et al. Transkingdom signaling based on bacterial cyclodipeptides with auxin activity in plants. *Proc. Natl. Acad. Sci. U S A*. **108**, 7253–7258 (2011). 21482761 10.1073/pnas.1006740108 PMC3084137

[32] Hoch, H. C., Staples, R. C., Whitehead, B., Comeau, J. & Wolf, E. D. Signaling for growth orientation and cell differentiation by surface topography in *Uromyces*. *Science* **235**, 1659–1662 (1987). 17795599 10.1126/science.235.4796.1659

[33] Cooper, B., Beard, H. S., Garrett, W. M. & Campbell, K. B. Benzothiadiazole conditions the bean proteome for immunity to bean rust. *Mol. Plant. Microbe Interact.* **33**, 600–611 (2020). 31999214 10.1094/MPMI-09-19-0250-R

[34] Bernsdorff, F. et al. Pipecolic acid orchestrates plant systemic acquired resistance and defense priming via Salicylic acid-Dependent and -Independent pathways. *Plant. Cell.* **28**, 102–129 (2016). 26672068 10.1105/tpc.15.00496 PMC4746677

[35] Klessig, D. F., Choi, H. W. & Dempsey, D. A. Systemic acquired resistance and Salicylic acid: Past, Present, and future. *Mol. Plant. Microbe Interact.* **31**, 871–888 (2018). 29781762 10.1094/MPMI-03-18-0067-CR

[36] Jirage, D. et al. Constitutive Salicylic acid-dependent signaling in cpr1 and cpr6 mutants requires PAD4. *Plant. J.* **26**, 395–407 (2001). 11439127 10.1046/j.1365-313x.2001.2641040.x

[37] Wang, W. et al. An Arabidopsis secondary metabolite directly targets expression of the bacterial type III secretion system to inhibit bacterial virulence. *Cell. Host Microbe*. **27**, 601–613e607 (2020). 32272078 10.1016/j.chom.2020.03.004

[38] Ngou, B. P. M., Ding, P. & Jones, J. D. G. Thirty years of resistance: Zig-zag through the plant immune system. *Plant. Cell.* **34**, 1447–1478 (2022). 35167697 10.1093/plcell/koac041 PMC9048904

[39] Cooper, B., Beard, H. S., Yang, R., Garrett, W. M. & Campbell, K. B. Bacterial immobilization and toxicity induced by a bean plant immune system. *J. Proteome Res.* **20**, 3664–3677 (2021). 34097416 10.1021/acs.jproteome.1c00232

[40] O’Leary, B. M. et al. Early changes in Apoplast composition associated with defence and disease in interactions between phaseolus vulgaris and the halo blight pathogen Pseudomonas syringae Pv. phaseolicola. *Plant. Cell. Environ.* **39**, 2172–2184 (2016). 27239727 10.1111/pce.12770 PMC5026161

[41] Freeman, B. C. & Beattie, G. A. Bacterial growth restriction during host resistance to Pseudomonas syringae is associated with leaf water loss and localized cessation of vascular activity in Arabidopsis Thaliana. *Mol. Plant. Microbe Interact.* **22**, 857–867 (2009). 19522568 10.1094/MPMI-22-7-0857

[42] Yamada, K., Saijo, Y., Nakagami, H. & Takano, Y. Regulation of sugar transporter activity for antibacterial defense in Arabidopsis. *Science* **354**, 1427–1430 (2016). 27884939 10.1126/science.aah5692

[43] Parniske, M., Ahlborn, B. & Werner, D. Isoflavonoid-inducible resistance to the phytoalexin Glyceollin in soybean rhizobia. *J. Bacteriol.* **173**, 3432–3439 (1991). 2045365 10.1128/jb.173.11.3432-3439.1991 PMC207956

[44] Kapadia, C. et al. Pseudomonas aeruginosa inhibits quorum-sensing mechanisms of soft rot pathogen Lelliottia Amnigena RCE to regulate its virulence factors and biofilm formation. *Front. Microbiol.* **13**, 977669 (2022). 36090086 10.3389/fmicb.2022.977669 PMC9450810

[45] Wu, L., Wu, H., Chen, L., Zhang, H. & Gao, X. Induction of systemic disease resistance in Nicotiana benthamiana by the cyclodipeptides cyclo (l-Pro-l-Pro) and cyclo (d-Pro-d-Pro). *Mol. Plant Pathol.* **18**, 67–74 (2017). 26836580 10.1111/mpp.12381 PMC6638238

[46] Minen, R. I. et al. Characterization of the cyclic dipeptide cyclo(His-Pro) in arabidopsis. *Plant Physiol.* **198** (2025). 10.1093/plphys/kiaf174 PMC12089766 40317191

[47] Cooper, B. & Yang, R. Genomic resources for Pseudomonas Savastanoi pv. phaseolicola races 5 and 8. *Phytopathology* **111**, 893–895 (2021). 33315475 10.1094/PHYTO-10-20-0462-A

[48] Cooper, B. & Yang, R. An assessment of acquirex and compound discoverer software 3.3 for non-targeted metabolomics. *Sci. Rep.* **14**, 4841 (2024). 38418855 10.1038/s41598-024-55356-3 PMC10902394

[49] Lee, J. et al. Quantitative proteomic analysis of bean plants infected by a virulent and avirulent obligate rust fungus. *Mol. Cell. Proteom.* **8**, 19–31 (2009). 10.1074/mcp.M800156-MCP200 18755735

